# Meta-Analysis of Structural and Functional Brain Abnormalities in Cocaine Addiction

**DOI:** 10.3389/fpsyt.2022.927075

**Published:** 2022-06-24

**Authors:** Jinghan Dang, Qiuying Tao, Xiaoyu Niu, Mengzhe Zhang, Xinyu Gao, Zhengui Yang, Miaomiao Yu, Weijian Wang, Shaoqiang Han, Jingliang Cheng, Yong Zhang

**Affiliations:** Department of Magnetic Resonance Imaging, First Affiliated Hospital of Zhengzhou University, Zhengzhou, China

**Keywords:** cocaine addiction, voxel-based morphometry, gray matter, meta-analysis, functional magnetic resonance imaging

## Abstract

**Background:**

Previous voxel-based morphometric (VBM) and functional magnetic resonance imaging (fMRI) studies have shown changes in brain structure and function in cocaine addiction (CD) patients compared to healthy controls (HC). However, the results of these studies are poorly reproducible, and it is unclear whether there are common and specific neuroimaging changes. This meta-analysis study aimed to identify structural, functional, and multimodal abnormalities in CD patients.

**Methods:**

The PubMed database was searched for VBM and task-state fMRI studies performed in CD patients between January 1, 2010, and December 31, 2021, using the SEED-BASE d MAP software package to perform two independent meta-groups of functional neural activation and gray matter volume, respectively. Analysis, followed by multimodal analysis to uncover structural, functional, and multimodal abnormalities between CD and HC.

**Results:**

The meta-analysis included 14 CD fMRI studies (400 CD patients and 387 HCs) and 11 CD VBM studies (368 CD patients and 387 controls). Structurally, VBM analysis revealed significantly lower gray matter volumes in the right superior temporal gyrus, right insula, and right retrocentral gyrus than in the HC. On the other hand, the right inferior parietal gyrus increased in gray matter (GM) volume in CD patients. Functionally, fMRI analysis revealed activation in the right temporal pole, right insula, and right parahippocampal gyrus. In the right inferior parietal gyrus, the left inferior parietal gyrus, the left middle occipital gyrus, and the right middle frontal gyrus, the degree of activation was lower.

**Conclusion:**

This meta-analysis showed that CD patients had significant brain GM and neural changes compared with normal controls. Furthermore, multi-domain assessments capture different aspects of neuronal alterations in CD, which may help develop effective interventions for specific functions.

## Introduction

Cocaine is an alkaloid that is produced biosynthetically by Erythroxylum coca, a shrub native to the Andean Highlands and northern parts of the Amazon in South America (1). This psychostimulant drug has become an essential part of the world drug scene and is also the world’s most trafficked drug after cannabis (resin or marijuana) (2). The worldwide prevalence of cocaine use was estimated at 0.3–0.4% of the population aged 15–64 years (between 13 and 20 million users) (3). Recent epidemiological data indicate that the prevalence of cocaine use is increasing (2).

Addiction is a chronic relapsing disorder characterized by the loss of inhibitory control over drug-seeking and taking, and maintenance of drug use despite negative consequences (4). Cocaine addiction (CD) is a worldwide public health problem, which has somatic, psychological, psychiatric, socio-economic, and judicial complications (5). It’s short half-life and strong dopaminergic precursor activity make it the most addictive of the psychostimulants (6). Numerous studies have shown that cocaine causes irreversible structural changes in the brain, heart, lungs, and other organs such as the liver and kidneys, and that many mechanisms are involved in the occurrence of these damages (7). Compared with the general population, cocaine use is associated with a significantly increased risk of schizophrenia (8) and may also induce transient psychotic symptoms such as paranoid beliefs and paranoia, hallucinations, and stereotyped actions (9). Psychosis develops during substance use and may not resolve even after withdrawal or withdrawal (10). Stroke risk appears to be significantly increased with cocaine use (11–13). The addictive nature of this drug can cause significant acute and long-term psychological effects in humans (14). It is worth mentioning that crack cocaine users have more family problems than other drug users, and previous research has shown that this population has higher rates of living on the streets and coming from broken homes (15). In light of the significant prevalence and negative consequences of CD, proposed diagnostic criteria have been included in the 5th edition of the Diagnostic and Statistical Manual of Mental Disorders (DSM-5) and have been widely used to diagnose addiction and evaluate its treatment (16).

With the development of modern imaging technology, functional magnetic resonance imaging, and voxel-based morphometric analysis are widely used to study the brain characteristics of CD. However, these studies often provide inconsistent results, in part because of the differences in the methods and sample characteristics of most studies and the limited number of samples. Therefore, the persistent neurological changes associated with CD are still largely unknown. It is of great significance to conduct meta-analysis to draw a consistent conclusion. In addition to functional neurological changes, structural markers such as gray matter volume are also important because they may be relatively stable over time and can be used as the basis for functional neural activity (17). Voxel-based morphometry (VBM) analysis is a standardized method for measuring the volume of gray matter, and has been widely used in the study of CD. Therefore, CD’s VBM study can provide additional information and complement the findings of fMRI research. In previous studies using VBM gray matter (GM) volume, almost identical reductions in insular and temporal GM volume compared with controls have been reported (18–21). However, studies using functional magnetic resonance (fMRI) reported more significant changes in prefrontal cortex function compared with controls (22–26).

To sum up, the purpose of this study is to explore the common GM abnormalities and functional deficits of CD individuals, which are very important for the development of specific intervention for CD or transdiagnostic treatment. We conducted two meta-analyses including all VBM and fMRI studies separately, and further performed a joint analysis between the two main meta-analyses. Based on previous studies, we hypothesized that the Insula in CD subjects would develop disorder-specific GMV abnormalities. As for fMRI, we hypothesized that CD patients exhibit abnormal under activation of the prefrontal cortex (PFC).

## Methods

### Inclusion of Studies for Meta-Analysis

We did our Meta-Analyses in accordance with PRISMA (Preferred Reporting Items for Systematic Reviews and Meta-Analyses) guidelines. Systematic and comprehensive searches of VBM and fMRI studies of CD from Jan 1, 2010, to Dec 31, 2021 was performed using the PubMed, Web of Science and Scopus database, combined with the following keywords: (“cocaine” or “cocaine related disorders” or “cocaine addiction” or “CD”) and (“voxel-based morphometry” or “VBM” or “gray matter” or “functional magnetic resonance imaging” or “fMRI”) and “brain.” Studies were included if the following inclusion criteria were met: (1) they reported whole brain results; (2) they compared CDs and HCs; (3) they were a task-related fMRI or VBM study; (4) they provided peak coordinates in Montreal Neurological Institute (MNI) or Talairach spaces; (5) the diagnoses of each study were based on DSM. (6) They used consistent thresholds in different regions. Studies were excluded if (1) the patient group included other diseases; (2) they did not use VBM; (3) peak coordinates were not reported; (4) only region of interest results were available; (5) inconsistent thresholds were applied in different regions. (6) they had fewer than 10 patients.

### Quality Assessment

Two investigators (Jinghan Dang and Qiuying Tao) independently assessed the quality of each included study using the Newcastle-Ottawa Scale (NOS) (27). NOS consist of three quality parameters for cohort studies: selection, comparability, and outcome, which are assigned with a maximum of four, two, and three stars, respectively. Therefore, nine stars reflect the highest quality. Studies with more than six stars are considered high quality (28). Any discrepancy was resolved through a joint revaluation of the original study with a third author (Xiaoyu Niu).

### Statistical Analyses

We performed a coordinate-based meta-analysis (29) using the anisotropic effect size version of the SDM software package (Version 5.15) for GM volumes between long-term cocaine use and healthy controls Variety. The SDM method has been well validated and described in detail in many studies. The data processing procedure is briefly summarized here (30). For each meta-analysis, AES-SDM converted peak coordinates to Hedge’s effect size and recreated voxel-level maps for each study based on an anisotropic unnormalized Gaussian kernel. Mean plots were then calculated using a random effects model, weighted to account for sample size, within-study variability, and between-study heterogeneity (31). Here are the steps: (1) *p*-values or *z*-values in some studies need to be converted to *t*-values online (32); (2) the peak coordinates are converted to normalized MNI space; (3) set the full width at half maximum (FWHM) to 20 mm as this will maintain a balance between sensitivity and specificity and other parameters including voxel *p* < 0.005, peak height threshold > 1 and cluster extent threshold > 10 voxels; (4) After excluding one study at a time, a jack-knife sensitivity analysis was performed by repeating the meta-analysis to verify the stability and reliability of the results. If a brain region survives most repetitions, we can conclude that the abnormality is stable (33). (5) To check for possible publication bias, the effect size at the peak point for each significant cluster of each study was extracted and funnel plots were constructed with the standard error of the effect as the vertical axis and the effect size as the horizontal axis (31). Additionally, Egger’s test, a regression of standard normal deviation (defined as effect size divided by its standard error) on precision (defined as the inverse of the standard error of effect size), was used to quantitatively test the asymmetry of each funnel plot. A funnel plot is considered asymmetric if the intercept of the regression deviates significantly from 0 (34, 35).

## Results

### Sample Characteristics of Included Studies

**FIGURE 1:**
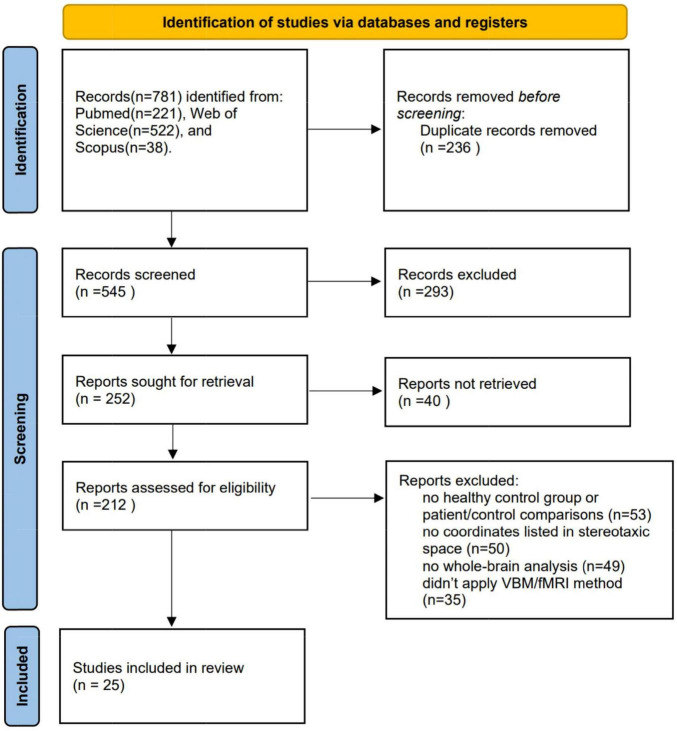
Procedure for including eligible studies in the meta-analysis. VBM, voxel- based morphometry; fMRI, functional magnetic resonance imaging.

As demonstrated in Figure 1, the final dataset contains 14 fMRI contrasts covering 400 CDs and 387 HCs, and 11 VBM contrasts covering 368 CDs and 387 HCs. See Table 1 for more demographic, clinical, and other characteristics.

**TABLE 1:** Sample characteristics of VBM and fMRI studies in CD group.

Study	Patients		Controls		Clinical characteristics		Task
	*N* (%male)	Age, year	*N* (%male)	Age, year	Diagnostic criteria	Duration of illness (years)	
**VBM studies in CD**							
Moreno-López et al. (21)	38 (100)	29.6	38 (100)	31.1	DSM-IV	4.1	NA
Barrós-Loscertales et al. (18)	20 (100)	33.3	16 (100)	33.4	DSM-IV	NA	NA
Meade et al. (65)	39 (62)	45.4	40 (60)	43.5	DSM-IV-TR	NA	NA
Rabin et al. (66)	28 (71)	49.6	45 (67)	39.4	DSM-IV/DSM-5	23.1	NA
Alia-Klein et al. (67)	40 (100)	45.0	42 (100)	39.0	DSM-IV	19.0	NA
Crunelle et al. (68)	30 (NA)	20–55	33 (NA)	20–55	Consumption of cocaine	NA	NA
Gardini and Venneri (19)	14 (NA)	31.1	24 (NA)	33.2	DSM-IV	13.4	NA
Hanlon et al. (20)	24 (71)	38.9	25 (48)	36.2	DSM-IV	11.1	NA
Ide et al. (69)	84 (65)	39.8	86 (55)	38.1	DSM-IV	18.0	NA
Matuskey et al. (70)	14 (29)	41.0	10 (50)	30.7	DSM-IV	21.0	NA
Yip et al. (71)	37 (68)	42.4	37 (76)	38.0	DSM-IV	NA	NA
**fMRI studies in CD**							
Asensio et al. (22)	32 (100)	36.6	26 (100)	28.4	DSM-IV	11.4	Visual discrimination task
Konova et al. (72)	21 (81)	43.1	21 (86)	38.9	DSM-IV	17.8	Monetary reward paradigm
Moeller et al. (73)	33 (85)	43.9	20 (90)	39.6	DSM-IV	14.3	Inhibitory control task
Bedi et al. (74)	22 (82)	52.9	19 (79)	52.7	DSM-IV	21.7	Social and non-social reward/threat processing
Moeller et al. (75)	2 1 (100)	43.2	17 (100)	32.6	DSM-IV	18.8	Stroop task
Verdejo-Garcia et al. (25)	18 (95)	34.3	18 (95)	31.2	DSM-IV-TR	3.6	Probabilistic reversal learning task
Ide et al. (76)	75 (67)	39.9	88 (56)	38.7	DSM-IV	18	Stop signal task
Konova et al. (72)	28 (50)	44.0	25 (56)	40.0	DSM-IV	15.5	Monetary reward task
Tobler et al. (24)	17 (76)	33.0	17 (71)	34.5	DSM-IV	8.4	Social reward task
Verdejo-Garcia et al. (77)	19 (95)	35.4	19 (95)	30.8	DSM-IV	4.8	Social decision-making task
Zhang et al. (78)	20 (85)	46.8	24 (79)	46.3	DSM-IV	18.8	Cue-induced cocaine craving task and Cue-induced food craving task
Mitchell et al. (79)	15 (40)	39.0	15 (47)	40.0	DSM-IV	11.1	Stroop task
Canterberry et al. (26)	20 (NA)	35.6	20 (NA)	34.9	DSM-IV-TR	17.8	Assess image recognition
Kaag et al. (23)	59 (NA)	31.4	58 (NA)	30.5	DSM-IV	12	Cue reactivity paradigm

### Quality Assessment

The 11 studies of VBM had an average NOS score of 6.36, and the 14 studies of fMRI had an average NOS score of 6.36. They were all of high quality (NOS score ≥ 6) (36) (Supplementary Table 1).

### Main Meta-Analyses for Functional Magnetic Resonance Imaging and Voxel-Based Morphometric Studies

**FIGURE 2:**
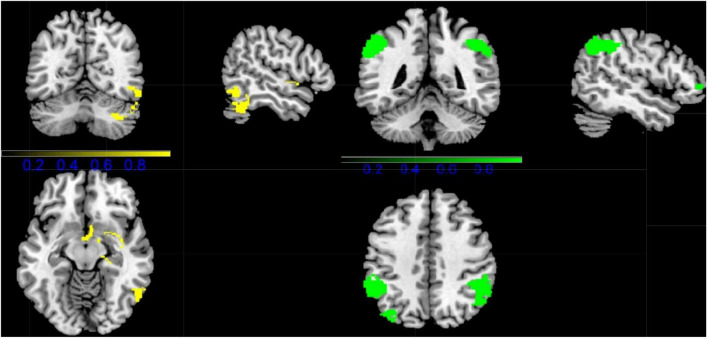
Results of Functional Magnetic Resonance Imaging (fMRI) for CD. (Yellow, CD increased; Green: CD decreased).

Combining all fMRI studies, CDs showed activation in the right inferior temporal gyrus (ITG.R), right insula (INS.R), right parahippocampal gyrus (HIP.R), and right temporal pole: superior temporal gyrus (TPOsup.R) compared to HCS. However, CDS activation was lower in the right inferior parietal gyrus (IPL.R), left inferior parietal gyrus (IPL.L), left middle occipital gyrus (MOG.L), and right middle frontal gyrus (MFG.L) (Figure 2 and Table 2).

**TABLE 2:** Results of all meta-analyses.

Meta-analysis	Region	MNI coord (x, y, z)	SDM-Z	P	Voxels	Cluster’ breakdown (voxels)	Jack-knife sensitivity	BA
**1. VBM RESULTS**								
1) CDs > HCs	R inferior parietal gyri	50, –52, 52	1.013	0.001723707	416	R inferior parietal gyri (305)	10 out of 11	40,39
						R angular gyrus (50)		
						R inferior parietal gyri (25)		
2) HCs > CDs	R temporal pole, superior temporal gyrus	32, 8, –26	–3.278	∼0	771	R temporal pole, superior temporal gyrus (98)	10 out of 11	38,28,34,36,30
						R parahippocampal gyrus (65)		
						R amygdala (64)		
						R parahippocampal gyrus (59)		
						R amygdala (26)		
						R fusiform gyrus (24)		
	R insula	42, –10, 8	–2.739	0.000149667	539	R rolandic operculum (194)	11 out of 11	48
						R insula (192)		
						R Heschl gyrus (93)		
						R insula (20)		
	R postcentral gyrus	22, –48, 62	–2.651	0.000237405	102	R superior parietal gyrus (32)	8 out of 11	7,2,5
						R postcentral gyrus (21)		
						R superior parietal gyrus (20)		
	(Undefined)	26, -66, –42	–2.356	0.001460493	95	(Undefined) (71)	9 out of 11	
**2. fMRI RESULTS**								
1) CDs > HCs	R inferior temporal gyrus	50, –44, –26	1.732	0.000283837	594	R inferior temporal gyrus (166)	13 out of 14	37,20
						R cerebellum, crus I (148)		
						R cerebellum, crus II (76)		
						R cerebellum, crus I (54)		
						R inferior temporal gyrus (43)		
	R insula	40, -6, -10	2.324	∼0	173	R lenticular nucleus, putamen (50)	12 out of 14	48
						R striatum (30)		
	R parahippocampal gyrus	14, –6, –22	1.495	0.001708210	92		11 out of 14	28
	R temporal pole, superior temporal gyrus	52, 4, –2	1.480	0.001893997	52		11 out of 14	48
	(Undefined)	2, 0, -12	1.768	0.000211596	47	(Undefined) (25)	13 out of 14	
2) HCs > CDs	R inferior parietal gyri	48, –44, 46	–2.356	0.000015497	948	R inferior parietal gyri (400)	13 out of 14	40,39
						R angular gyrus (213)		
						R supramarginal gyrus (185)		
						R angular gyrus (38)		
						R inferior parietal gyri (36)		
						Right superior longitudinal fasciculus III (26)		
	L inferior parietal gyri	–50, –44, 52	–2.270	0.000030994	826	L inferior parietal gyri (570)	13 out of 14	40,39
						L supramarginal gyrus (68)		
						L inferior parietal gyri (32)		
						L superior longitudinal fasciculus III (25)		
	L middle occipital gyrus	–44, –74, 32	–2.022	0.000221908	318	L angular gyrus (65)	13 out of 14	39,7,19
						L middle occipital gyrus (59)		
						L inferior parietal gyri (42)		
						L middle occipital gyrus (37)		
						L angular gyrus (25)		
						L angular gyrus (21)		
	R middle frontal gyrus	40, 44, 0	–1.797	0.001063108	137	R middle frontal gyrus, orbital part (25)	12 out of 14	47,46,45
						R inferior frontal gyrus, triangular part (20)		
**3. Multimodal analysis**								
	R inferior parietal gyri	48, –56, 50	1.000		265	R inferior parietal gyri (195)		40,39
						Right angular gyrus (38)		

**FIGURE 3:**
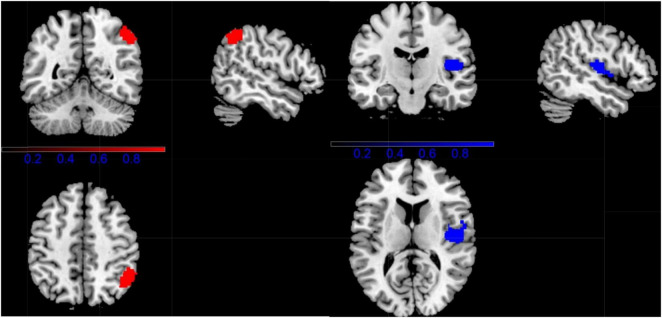
Results of Voxel-Based Morphometry (VBM) for CD. (Red, CD increased; Blue, CD decreased).

Regarding the VBM study, CD showed significantly lower gray-matter volume in right temporal pole: superior temporal gyrus (TPOsup.R), right insula (INS.R), and right postcentral gyrus (POCG.R), compared with HC. While people with CD showed increased GM volume in right inferior parietal gyri (ILF.R) (Figure 3 and Table 2).

### Multimodal Voxel-Based Morphometric and Functional Magnetic Resonance Imaging Analyses

**FIGURE 4:**
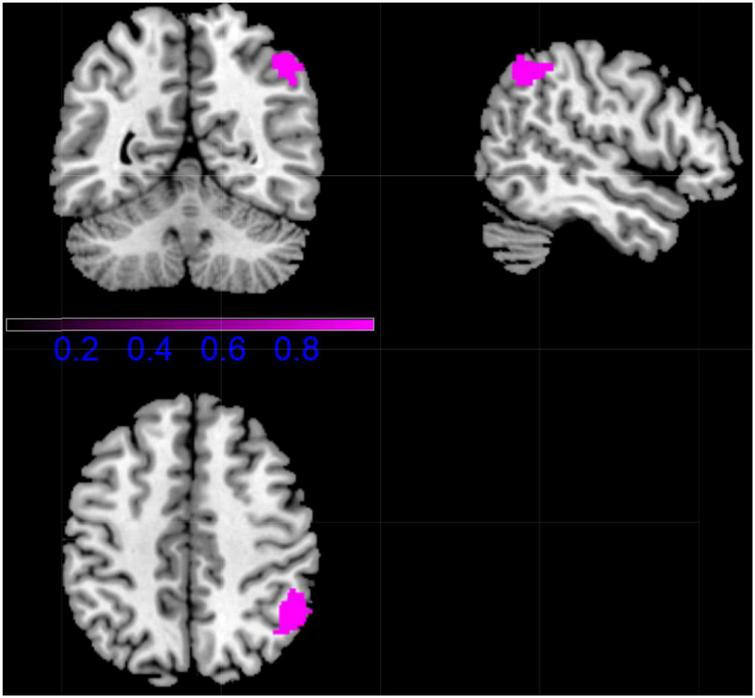
Results of Multimodal VBM and fMRI Analyses. (violet, CD increased).

In patients with CD, the Right inferior parietal (excluding supramarginal and angular) gyri was increased in volume and decreased in function connection relative to controls (MNI coordinates, 48, –56, 50, 265 voxels) (Figure 4 and Table 2).

### Reliability Analyses

To assess the reliability of the findings, Jackknife sensitivity analysis was performed. In major fMRI meta-analyses these results were highly reproducible, as at least 11 of the 14 combinations could be identified. For the VBM meta-analysis, changes in the right insula were preserved in all study combinations. Furthermore, the other results were remarkably robust as at least 8 of the 11 combinations were identifiable.

### Publication Bias

Egger’s tests were performed to examine potential publication bias. The results of the Egger tests were non-significant (*P* > 0.05 for all comparisons, Bonferroni corrected), suggesting that there was no publication bias.

## Discussion

The purpose of our meta-analysis is to explore the changes of brain GM and functional abnormalities between CD and HC. The main fMRI Meta-analysis showed that CD was related to the overactivation of ITG.R, INS.R, HIP.R, and TPOsup.R, but to the insufficient activation of IPL, MOG.L, and MFG.L. In addition, VBM meta-analysis showed that the gray matter volume of CD in TPOsup.R, INS.R, and PoCG.R decreased, while that of IPL.R increased. Through multimodal analysis, the gray matter volume of IPL.R was abnormally increased, but the function was not activated. The whole brain jack-knife sensitivity analysis of the system provides a reliable result.

According to our results, neural changes in the right insula were present in both the main VBM and fMRI meta-analyses. Previous studies have found that the gray matter value of the insular cortex is lower in drug-dependent patients (37–40). The insula is connected to several regions of the brain, such as the orbitofrontal cortex, frontal opercular structures, lateral premotor cortex, somatosensory area, parietal lobe, superior temporal sulcus, cingulate gyrus, amygdala, peri-olfactory, and entorhinal cortex. The insula projects and provides cortical input to components of the ventral striatum, a structure that plays a major role in addiction. This structure of the ventral striatum plays a role in the formation of stimulus-drug associations (41). Abnormal volumetric development of insular gray matter may lead to abnormal input to the ventral striatum leading to facilitation of addictive behaviors. Addiction, on the other hand, results from an imbalance between the unconscious impulse system and the conditioning system of conscious and cognitive control (42). When the balance is disrupted, the inhibitory function does not work, which results in people being unable to help with medication (43). Insula, the structural basis of the reflex system, which are responsible for impulsive control, decision making, and emotional regulation (44). GM atrophy of the insula was found in our meta-analysis, which may suggest that this abnormality contributes to poor impulse control, manifested by constant drug seeking and repetitive behaviors. In a recent review, Naqvi and Bechara (45) reviewed the existing literature on the role of the insula in drug addiction. Within their theoretical framework, it has been suggested that the insula modulates the reciprocal sensory effects of drugs, which then become available for consciousness, memory, and executive function, supporting a central role for this neural structure in addiction. Enhanced function of the insula in CD may indicate that they are accustomed to cocaine stimuli and insensitive to other conventional stimuli. Taken together, fMRI and VBM may reflect different aspects of neural alterations, with evidence focusing on the important role of the insula in CD.

The right middle frontal gyrus is less active in CD compared to HC, and the dmPFC in the supplementary motor area plays a key role in performance monitoring and cognitive control (46) and is associated with impaired inhibitory control in addicts (47). Our results show that attenuated PFC responses to stimuli fit the Impaired Response Inhibition and Significant Attribution (IRSA) model (48, 49). The IRSA model argues that drug dependence is mediated by dysfunction of the ACC and PFC, which involves a marked response to drug-related reward and is attributed to a high response to drug-related reward, and a markedly high response to non-drug-related stimuli Impairment (26), therefore, inactivation of the PFC in cocaine addicts may indicate that they are accustomed to the associated rewards brought on by cocaine and are insensitive to other non-drug rewards. Clinically, anhedonia is an important factor in cocaine relapse (50). Recent studies have demonstrated that Repetitive transcranial magnetic stimulation (rTMS) appears to have a unique therapeutic application for directly targeting and remodeling dysfunction in brain circuits altered by chronic cocaine exposure (51). Moreover, continuous rTMS regimen is safe and feasible in CD patients as a potential treatment (52). Our results may provide research directions for this method of brain stimulation.

According to the primary meta-analysis and multimodal analysis, the GM of the right inferior parietal gyrus increased but the activity decreased, and the activity of the left inferior parietal gyrus decreased. The bilateral inferior parietal lobules (IPL) mainly include the supramarginal and angular gyri. The inferior parietal lobule is a core node of the default mode network, a group of brain regions preferentially involved in mind formation (53). These regions were strongly deactivated in the goal orientation task compared to resting or passive baselines. It is thought to be involved in internal mental states that become salient when people do not engage in external interactions (53) and it forms the most consistent rest-state network (54). Inactivation of the inferior parietal cortex, including bilateral AGs, is highly reliable (55) and consistent across tasks, paradigms, subjects and studies (56, 57). Disruptions in connections between the DMN and cortical areas involved in executive function, memory and mood may be critical to drug use, research has shown (58). These findings suggest that CDs may lead to poor impulse control by reducing neural activity in the IPL and predispose people to some degree of addiction. As for the supramarginal gyrus, this may be an area involved in the visual memory system. In particular, these regions have been shown to be involved in the processing of visually presented stimuli and the extraction of spatial locations (59, 60). Evidence from these studies suggests that the right inferior parietal lobe, particularly the supramarginal gyrus, appears to play a role in processing visually presented information. Importantly, visual memory ability has been shown to be associated with treatment engagement as well as substance relapse (61). Whether the increase in GM in the inferior parietal gyrus of CD has an effect on visual memory ability can be a direction for future research.

In addition, previous studies have shown that some individuals with personality disorders, mainly those with social cognitive deficits, also have reduced temporal pole volume, such as those with cocaine-dependent personality disorders (62). And the temporal pole with right dominance is involved in various functions of social cognitive network, mainly emotion processing, empathy, and insight, which is consistent with our results. Furthermore, the superior temporal gyrus is implicated in impulsivity and craving (63) and is involved in the regulatory control of reward-seeking behavior (64), an important component of the addiction process. However, the exact mechanism of temporal pole abnormalities in CD-related diseases remains to be fully explored, and more attention should be paid in future studies.

### Limitation

This meta-analysis has certain limitations. First, a method based on peak coordinates was used in this study, rather than raw statistical brain maps, so it may be difficult to detect some results with small or moderate effects. Second, the heterogeneity of VBM research methods cannot be avoided, such as differences in MRI machines, slice thicknesses, preprocessing schemes (traditional or optimized), smoothing kernel sizes, and statistical thresholds may be responsible for inconsistent results. Third, this study focuses on the findings of task-based fMRI and VBM. Although studies using other techniques (e.g., diffusion tensor imaging, resting-state fMRI) may also provide valuable information on the neural mechanisms of CD, due to insufficient numbers of studies or limitations of coordinate-based methods, these Meta-analyses of modalities; therefore, future systematic reviews and meta-analyses of other modalities of CD are encouraged. Longitudinal studies could be conducted in the future to explore whether these brain regions may be potential neural targets for the treatment of cocaine addiction.

## Conclusion

In conclusion, possibly due to the limitations of the current meta-analysis, the results of this study showed that cocaine addicts had increased GM volume in the right inferior parietal gyrus and significantly decreased GM volume in the right superior temporal gyrus and right insula. There was increased activation in the right insula and right inferior temporal gyrus, and decreased activation in the bilateral inferior parietal gyrus and right middle frontal gyrus. The main evidence from brain function and gray matter volume comes together, suggesting that CD is associated with core neural changes in the right inferior parietal gyrus. In the future, we hope to further study the relationship between the right insula and the right inferior parietal gyrus and cocaine dependence. Future research using multimodal, multidomain research will further demonstrate and complement our foundation.

## Data Availability Statement

The raw data supporting the conclusions of this article will be made available by the authors, without undue reservation.

## Author Contributions

JD and YZ designed the experiment. JD, QT, and ZY performed the experiment. XN, XG, MZ, ZY, MY, WW, JC, SH, and YZ modified the experiment and manuscript. All authors contributed to the article and approved the submitted version.

## Conflict of Interest

The authors declare that the research was conducted in the absence of any commercial or financial relationships that could be construed as a potential conflict of interest.

## Publisher’s Note

All claims expressed in this article are solely those of the authors and do not necessarily represent those of their affiliated organizations, or those of the publisher, the editors and the reviewers. Any product that may be evaluated in this article, or claim that may be made by its manufacturer, is not guaranteed or endorsed by the publisher.
